# Sei-1 promotes double minute chromosomes formation through activation of the PI3K/Akt/BRCA1-Abraxas pathway and induces double-strand breaks in NIH-3T3 fibroblasts

**DOI:** 10.1038/s41419-018-0362-y

**Published:** 2018-03-01

**Authors:** Xing Tian, Chang Liu, Xin Wang, Fei Wang, Liqun Wang, Lu Xu, Jinfa Ma, Yating Gao, Yantao Bao, Falin Wang, Luyao Sun, Junni Wei, Chuwen Lin, He Zhang, Gang Zhu, Xinyuan Guan, Songbin Fu, Chunyu Zhang

**Affiliations:** 1 Laboratory of Medical Genetics, Harbin Medical University, 150081 Harbin, China; 2 The Department of Otolaryngology, Heilongjiang provincial hospital, 150081 Harbin, China; 3 Obstetrics Department, Fourth Affliated Hospital, Harbin Medical University, 150081 Harbin, China; 4 Department of Clinical Oncology, Faculty of Medicine, The University of Hong Kong, Hong Kong, China; 5 Key Laboratory of Medical Genetics, Harbin Medical University, Heilongjiang Higher Education Institutions, 150081 Harbin, China

## Abstract

*Sei-1* is a potential oncogene that plays an important role in promoting genomic instability. Double minute chromosomes (DMs) are hallmarks of gene amplification and contribute to tumorigenesis. Defects in the DNA double-strand break (DSB) repairing pathways can lead to gene amplification. To date, the mechanisms governing the formation of DMs induced by *Sei-1* are not fully understood. We established DMs induced by *Sei-1* in the NIH-3T3 cell line. RNA-sequencing was used to identify key characteristics of differentially expressed genes. Metaphase spreads were used to calculate DM numbers. Immunofluorescence was employed to detect γH2AX foci. Western blot and Akt pathway inhibition experiments were performed to reveal the role of the PI3K/Akt/BRCA1-Abraxas pathway in Sei-1-induced DMs. Luciferase reporter assay was employed to explore the regulatory mechanisms between *Sei-1* and *BRCA1*. DM formation was associated with a deficiency in DSB repair. Based on this finding, activation of the PI3K/Akt/BRCA1-Abraxas pathway was found to increase the DM population with passage in vivo, and inhibition resulted in a reduction of DMs. Apart from this, it was shown for the first time that *Sei-1* could directly regulate the expression of *BRCA1*. Our results suggest that the PI3K/Akt/BRCA1-Abraxas pathway is responsible for the formation of DMs induced by *Sei-1*.

## Introduction

Sei-1 (also known as TRIP-Br1 and SERTAD1) is a member of the TRIP-Br family, which acts as a nuclear factor that regulates the cell cycle by interacting with cyclin-dependent kinase 4 (CDK4) and E2F/DP-1^1^. As an identified oncogene, *Sei-1* is located in a frequent amplification region, 19q13.1, in ovarian cancer^2,3^ and is associated with mechanisms that lead to chromosomal instability, such as increasing the number of chromosomes, and double minute chromosomes (DMs) and micronuclei formation^4,5^. In addition, Sei-1 also inhibits apoptosis by stabilizing the X-linked inhibitor of apoptosis protein^6^ and by promoting metastasis through AKT or ILK modulation in HER2/neu-suppressed or expressing cancer cells^7^. However, few reports about the mechanisms that govern the formation of DMs and Sei-1 have been published so far, and as a result, further exploration in this field is needed.

DMs are paired, circular, acentric, and self-replicating DNA and are the cytogenetic hallmark of extrachromosomal gene amplification^8,9^. DMs can be detected in many tumors, such as acute myeloid leukemia^10^, ovarian carcinoma^11,12^, glioblastoma multiforme^13,14^, and colonic carcinoma^15,16^. Moreover, genes located on DMs can play crucial roles in the development of cancer, such as *DHFR* amplification in methotrexate resistance^17^ and *MYC* promotion of proliferation and invasion^18^. Therefore, the study of the DM formation is important for exploring the mechanisms underlying the development of tumors.

DNA double-strand breaks (DSBs) are a form of DNA damage that can lead to genomic instability or apoptosis if not appropriately repaired^19,20^. When a DSB occurs, it can initiate two main repair mechanisms, homologous recombination (HR) and classical non-homologous end joining. In addition, alternative end joining and single-strand annealing have been found to take part in DSB repair under certain conditions^20,21^. Breast cancer type 1 susceptibility protein (BRCA1) is recruited to the sites of DSBs with the help of the BRCA1-Abraxas (BRCA1-A) complex to effectively repair DNA. The BRCA1-A complex is composed of BRCA1 and Abraxas (also named FAM175A, CCDC98, and Abra1) as well as other components, including Rap80, BRCC36, BRE, and NBA1^22,23^. Abraxas possesses the domains necessary to interact with the BRCT domains of BRCA1 and other components, which reveals the role of central adaptor protein^24^. The BRCA1-A complex plays important roles in maintaining genomic integrity during the DNA damage response.

Previous studies have shown that Sei-1 not only transforms the NIH-3T3 passage in vivo model to possess characteristics of cancerous formations but it also increases the incidence of genomic instability^3,4^. Moreover, DMs can appear during the passages in vivo in this model, which has aroused interest in exploring how these DMs are produced during this process. Although we previously reported the role of the met pathway in the promotion of the formation of DMs induced by Sei-1 in NIH-3T3 murine fibroblasts^5^, deep-seated and unknown mechanisms are also involved. Therefore, we selected cells from different passages in vivo with high and low DM populations for RNA-sequencing (RNA-seq) to explain the process governing the formation of DMs.

## Results

### Sei-1 promotes DM formation with increasing passage number in vivo

**Fig. 1:**
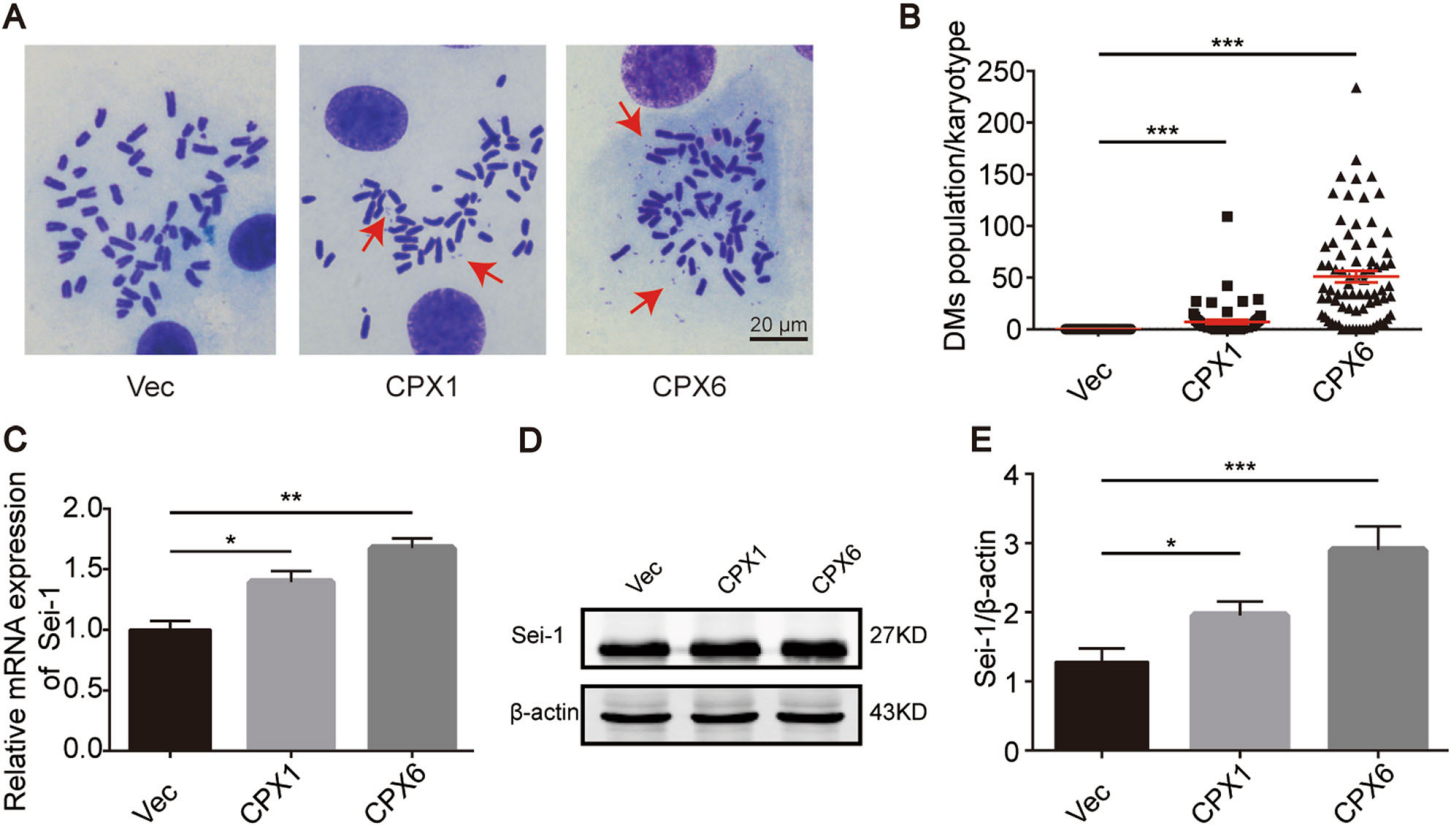
Sei-1 overexpression promotes the formation of DMs during in vivo passages . **a** Metaphase spread images of Vec, CPX1, and CPX6 samples. Red arrows represent the DMs. **b** Quantification of DMs in Vec, CPX1, and CPX6 samples; *n* = 100 from three independent experiments for each group. **c** Quantitative real-time PCR results of Sei-1 relative mRNA expression, *n* = 3. **d** Western blot results show increases in Sei-1 expression from the Vec, CPX1, and CPX6 samples during in vivo passages. **e** Quantification of the protein levels in **d**, *n* = 3. *P < 0.05; **P < 0.01; ***P < 0.001

To investigate the relationship between Sei-1 and the formation of DMs, we established the Sei-1-transformed NIH-3T3 model as previously described^5^. Consistent with previous results, the Sei-1-transfected group appeared to generate xenografts while the vector group failed. The primary cell culture from the original generation in vivo was named CPX1, and the sixth generation in vivo passage was named CPX6. The mean number of DMs for Vec, the control group transfected with *null*-vector-NIH-3T3 cells, and CPX1 and CPX6 was measured as ~0, 10, and 50, respectively (Fig. 1a, b). Next, quantitative real-time PCR (qPCR) was used to quantify *Sei-1* mRNA expression and showed that relative mRNA expression increased with the increasing number of DMs (Fig. 1c). In addition, the Sei-1 protein expression also increased (Fig. 1d, e). These results indicate that Sei-1 acts as a promoter of DM formation in vivo.

### DM generation is related to the occurrence of DSBs

**Fig. 2:**
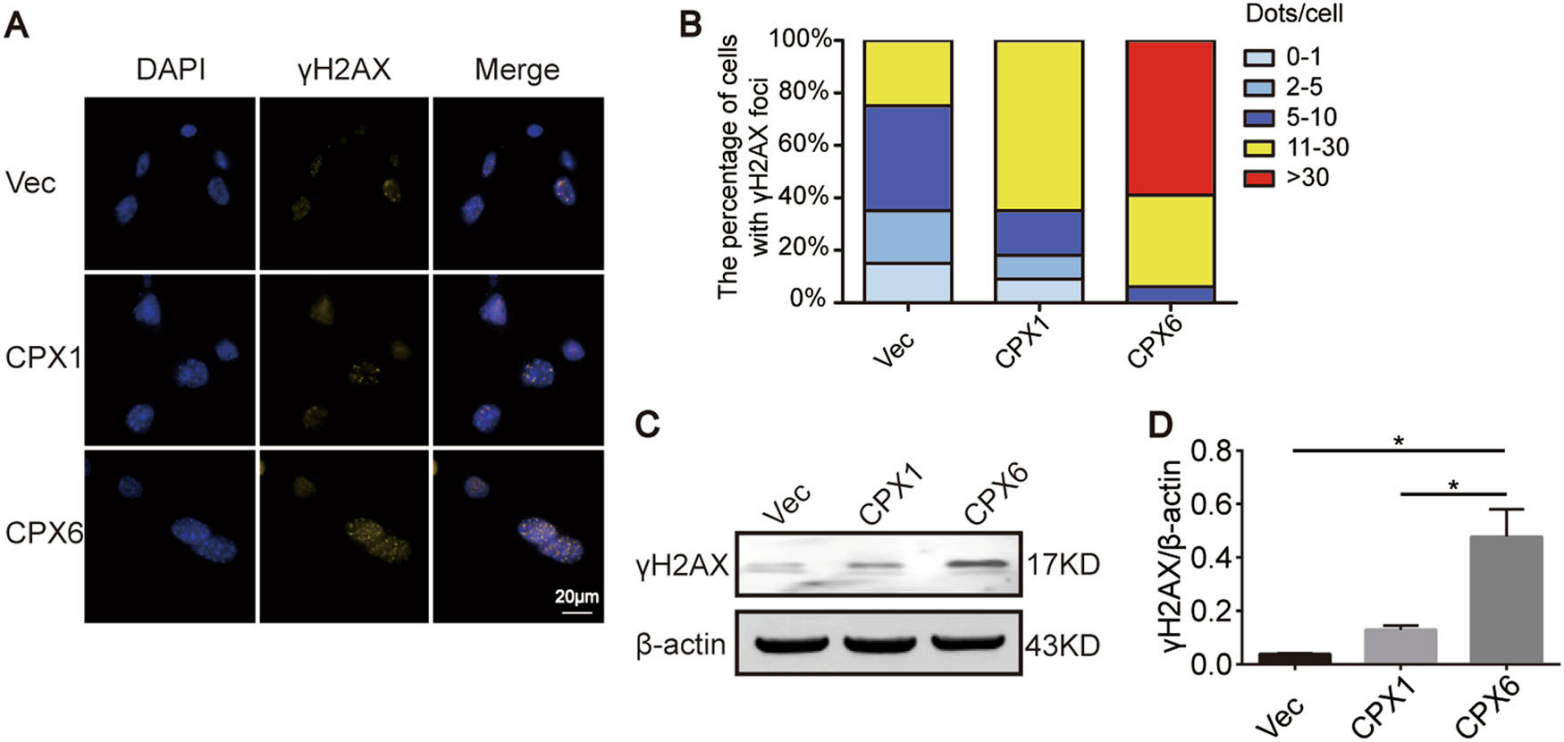
DSB occurrence is related to DM formation . **a** IF shows increases of γH2AX foci in the Vec, CPX1, and CPX6 samples. Yellow: γH2AX; blue: DAPI; magnification ×1000. **b** The percentage of γH2AX foci in A, *n* = 30. **c** Western blot shows increases of γH2AX. **d** Quantification of the protein levels in **c**, *n* = 3. *P < 0.05

The gene amplification process is associated with DSBs. Furthermore, DMs, which serve as one form of gene amplification, might be strongly related to DSBs. To explore this concept, γH2AX^3,25,26^ was selected as a marker to verify the existence of DSBs in this model. Immunofluorescence (IF) showed that the number of γH2AX foci increased significantly as the population of DMs increased (Figs. 1a, b and 2a, b). Western blot analysis also indicated that γH2AX protein levels correlated with the IF results (Fig. 2c, d). These results suggest that DM formation is inseparable from DSBs.

### *BRCA1* was selected as a pivotal gene from the RNA-seq data

**Fig. 3:**
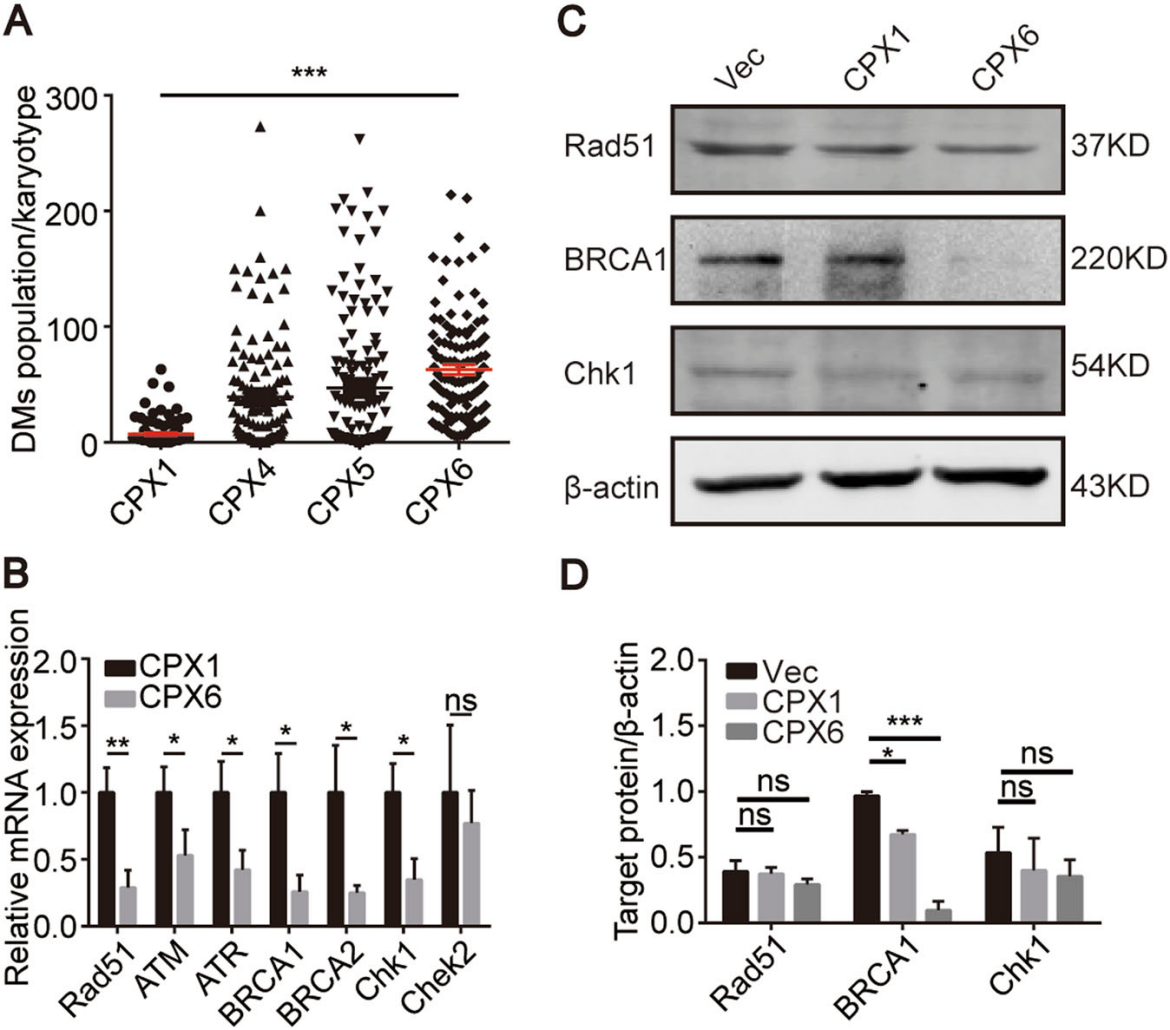
BRCA1 is a key DEG . **a** The increase in the number of DMs in CPX1, CPX4, CPX5, and CPX6 for each group. *n* = 100 from three independent studies. **b** Quantitative real-time PCR results of relative mRNA expression of the genes in Table 1, *n* = 3. **c** Western blot results show decreases of Rad51, BRCA1, and Chk1 levels. **d** Quantification of the protein levels in **c**, *n* = 3. *P < 0.05; **P < 0.01; ***P < 0.001

The DMs were counted in all of the primary cells, including CPX1, CPX4, CPX5, and CPX6, from each in vivo passage. These data suggest that the DM population increased gradually as the number of in vivo passages increased (Fig. 3a). CPX1 (C1), which had the lowest count, and CPX6 (C2), which had the highest count, were chosen for the RNA-seq experiments. In order to screen the differentially expressed genes (DEGs), the expression of all genes (*P* < 0.05) were analyzed and shown in supplementary figure 1A, and the detail information of RNA-seq data was supplied in the SRA database (SRP130842). Then, the enrichment analysis based on the DEGs showed that the DEGs were significantly associated with changes of DNA conformation, DNA metabolism, and chromosome-related regulation in Gene Ontology terms and PI3K/Akt signaling pathway was significantly enriched based on Kyoto Encyclopedia of Genes and Genomes database (Supplementary Figure 1B). In our previous study, we found that met could activate the PI3K/Akt signaling pathway and was exactly enriched in the abovementioned pathway in RNA-seq data (data not shown)^5^. All of these suggested that DEGs associated with changes of DNA structures and PI3K/Akt pathway could play essential roles in the process of DM formation. Therefore, many DEGs that were involved in DSB repair (Table 1) were filtered according to the analysis of RNA-seq data and the detection of DSBs. Almost all genes were downregulated except for *ATM*, while *BRCA1*, *Rad51*, and *Chek1* reached significance (*P* < 0.05). The qPCR was used to verify the accuracy of the RNA-seq data. All genes listed in the table were downregulated and only *BRCA1*, *Rad51*, and *Chek1* reached the same degree of significance as the original data (Fig. 3b). Hence, BRCA1, Rad51, and Chek1 protein expression were subsequently measured. BRCA1 was the only protein to show results that were consistent with the RNA-seq data, including the expression tendency of protein and statistical significance (Figs. 3c, d). These results indicate that there are defects in the DSB-associated repair pathway and *BRCA1* is a key gene in this process. As a result, BRCA1 was the focus of subsequent experiments.

**Table 1:** DSB-associated gene expression from RNA-seq data

Gene	Read count		*p* value	*q* value	Log2.Fold_change
	C1	C2			
*BRCA1*	56.15029303	27.91804213	0.00076613	0.010053	−1.0081
*Chek1*	24.362124	9.603806492	0.0062685	0.057512	−1.343
*Rad51*	57.55918695	22.03664125	1.75E−05	0.00037981	−1.3851
*ATM*	32.46326403	46.08338154	0.19742	0.67448	0.50544
*ATR*	37.86402405	28.14138646	0.1525	0.5796	−0.42813
*BRCA2*	17.75793376	10.90664846	0.15062	0.57492	−0.70326
*Chek2*	11.47661504	8.375412638	0.40965	0.96019	−0.45447

Note: C1, CPX1; C2, CPX6; *q* value, the lower value, the more difference between C1 and C2; log 2.Fold_change, C2/C1; A significant difference is defined as *P* < 0.05

*DSB* double-strand break, *RNA-seq* RNA-sequencing

### The PI3K/Akt/BRCA1-A complex pathway is activated when the DM population increases

**Fig. 4:**
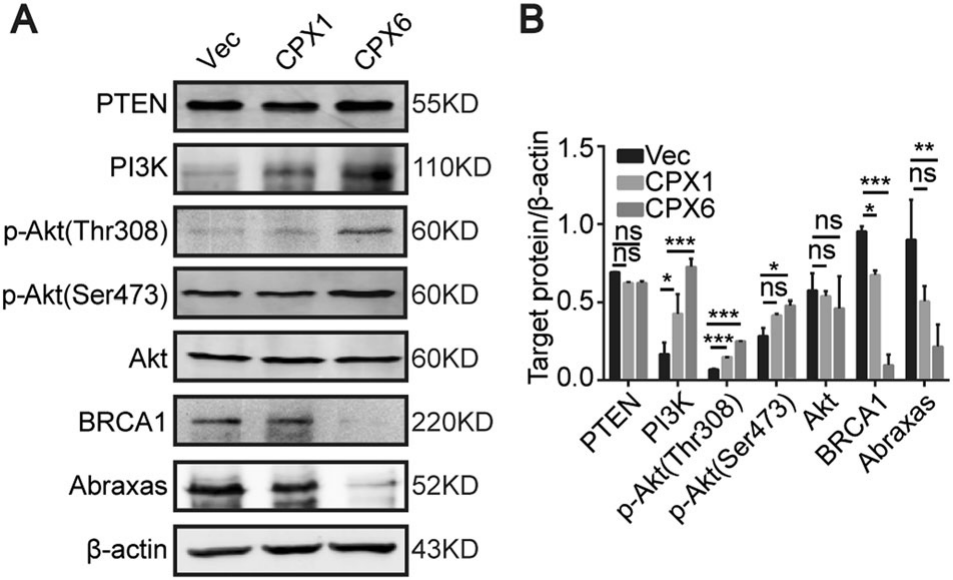
The PI3K/Akt/BRCA1-A complex pathway is activated as the population of DM increases . **a** Western blot results show increases in the PI3K/Akt pathway without the influence of PTEN as well as the reduction of BRCA1-A complex from Vec, CPX1, and CPX6 samples as DMs increase. **b** Quantification of the protein levels in **a**, *n* = 3. *P < 0.05; **P < 0.01; ***P < 0.001

BRCA1 is involved in many cellular processes including cell cycle checkpoints^27,28^ and DNA damage repair^28,29^. Furthermore, BRCA1 deficiency in breast epithelial cells could disable HR mechanisms for DNA damage, resulting in genomic instability and increased breast cancer risk. BRCA1-deficient mouse embryonic stem cells showed disabled DSB repair via HR^29^. Therefore, the role of BRCA1 in maintaining genomic integrity occupies an important position for DSBs. Previous studies demonstrated that BRCA1 is negatively regulated by the Akt signaling pathway^30,31^. Based on this finding and the enrichment analysis of RNA-seq data, we questioned whether the generation of DMs in this model was related to the activation of the Akt signaling pathway and loss of BRCA1 function. Thus, we measured the proteins involved in the Akt pathway and BRCA1 with immunoblot. We found a prominent increase in PI3K, phospho-Akt (Ser473), and phospho-Akt (Thr308), while there was no change in total Akt, and a significant reduction in BRCA1 (Fig. 4). BRCA1 has been shown to possess BRCT domains that interact with the pSPxF motif of Abraxas (A), Bach1/FancJ (B), and CtIP (C) to form three kinds of complexes in different phospho-dependent manners^23,24^. The BRCA1-A complex plays essential roles in maintaining genomic stability. Deficiencies in either BRCA1^32,33^ or Abraxas^22^ increased tumor incidence in mouse models. A decreased protein level of Abraxas was detected (Fig. 4), which is consistent with changes in BRCA1 expression. PTEN acts as a negative regulator for the PI3K/Akt pathway^34^, which suggests that it may regulate the PI3K/Akt pathway in the process of the formation of DMs. No changes in PTEN expression were observed (Fig. 4). Since Sei-1 is also a transcription factor, it would be interesting to explore its relationship with the pivotal *BRCA1*. Luciferase reporter assay showed that the luciferase activity was significantly upregulated in *Sei-1*-co-transfected and pGL3-BRCA1 promoter-co-transfected group in 293T cells (Supplementary figure 2). These findings suggest that the PI3K/Akt/BRCA1-A complex signaling pathway is activated as the DM population increases and PTEN has no influence on DM formation (Figs. 1a, b and 4). This also indicates that Sei-1 can also directly regulate the expression of *BRCA1*.

### DM population is reduced through the inhibition of the PI3K/Akt/BRCA1-A complex pathway

**Fig. 5:**
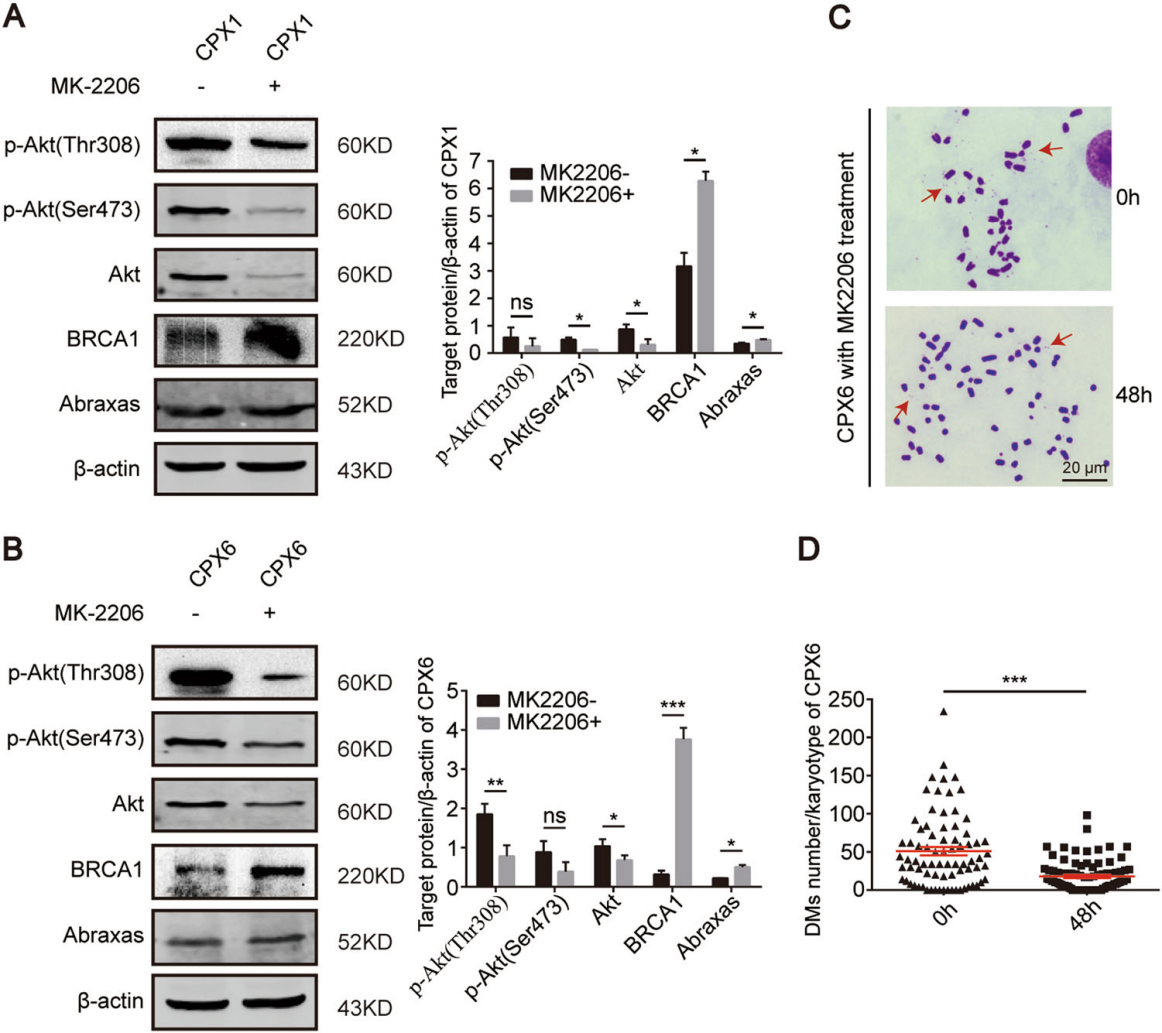
Inhibition of the PI3K/Akt/BRCA1-A complex pathway can result in the loss of DMs . **a**, **b** Western blot results indicate effective inhibition of the Akt pathway with the MK2206 inhibitor at 10 µM and increases of the downstream BRCA1-A complex in CPX1 or CPX6. Quantification of the protein levels, *n* = 3. **c** Metaphase spread images of CPX6 after inhibition of the Akt pathway. Red arrows indicate DMs. **d** Quantification of DMs in C, *n* = 100 from three independent experiments. *P < 0.05; **P < 0.01; ***P < 0.001

To explore the main function of the PI3K/Akt/BRCA1-A complex pathway in the production of DMs in Sei-1-transformed NIH-3T3 cells, MK2206 (an allosteric Akt inhibitor)^35,36^ was employed to inhibit the Akt signaling pathway. Western blot analysis showed decreasing levels of phospho-Akt (Ser473) and phospho-Akt (Thr308) in addition to significant decreases in total Akt. BRCA1 and Abraxas showed dramatic increases in expression after 10 µM drug treatment for 48 h (Fig. 5a, b). PI3K was not detected because MK2206 only inhibits a downstream molecule of the PI3K/Akt pathway. Furthermore, metaphase spreads were prepared to quantify the number of DMs at 0 and 48 h after 10 µM drug. CPX6 was chosen for counting DMs due to insufficient numbers of CPX1 samples. This revealed that the mean number of DMs decreased from ~50 to 20 after undergoing drug treatment (Fig. 5c, d). Collectively, these results demonstrate that the PI3K/Akt/BRCA1-A complex signaling pathway plays a vital role in the Sei-1-induced DM formation.

## Discussion

RNA-seq, which is a deep-sequencing technology, is used to map and quantify transcriptomes and has revealed new transcriptomic insights into gene and exon boundaries, transcript complexity, novel transcription mechanisms, and DEG identification^37,38^. In the present study, we aimed to sift through the DEGs related to DM formation, and in doing so identified *BRCA1* as an essential gene. This was done using RNA-seq, which differs from the previous study we performed^5^. In addition, we found that most genes associated with the DSB repair pathway were downregulated, which indicated that a defect existed in repairing DSBs. Gene amplification increases when the factors involved in the occurrence of DSBs, such as IR, DNA synthesis inhibitors, and restriction endonucleases, are added. Defects in either HR-relevant genes such as *ATM*^39^, *Rad51D*^40^, and *Rad54*^41^ or in NHEJ interrelated *DNA-PKcs*^42^ can enhance the predisposition of gene amplification mechanisms that include homogeneous staining regions and DMs. Consequently, these phenomena hint that Sei-1 may act as a factor that promotes DSBs and that there is a relationship between the production of DSBs and DMs in the Sei-1-transformed NIH-3T3 model.

**Fig. 6:**
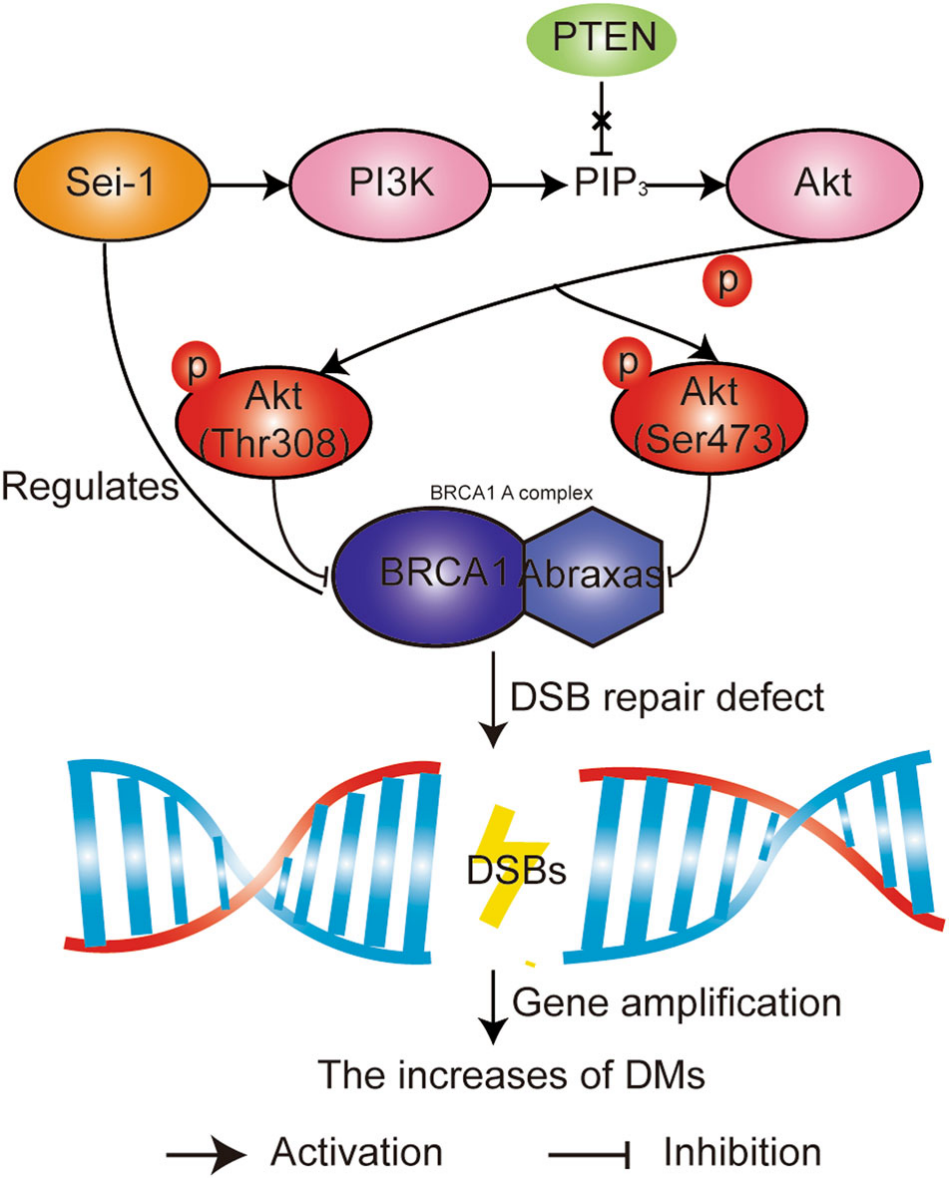
Schematic overview of the PI3K/Akt/BRCA1-A complex pathway in the Sei-1-transformed NIH-3T3 cell model . Sei-1 activates the PI3K/Akt pathway independent of PTEN and inhibits the expression of the BRCA1-A complex to result in DSBs, leading to the formation of DMs in vivo. Sei-1 can also target the promoter and regulate the expression of *BRCA1*

The PI3K/Akt pathway participates in many aspects of tumor biology, including cell proliferation, migration, invasion, metastasis, and survival. Activation of Akt, with the loss of PTEN, can impair Chk1 through ubiquitination, phosphorylation, and reduction of nuclear localization to mediate genomic instability^43^, but its relationship with the generation of DMs is still unknown. We found that the activation of PI3K/Akt promoted the appearance of DMs in a PTEN-independent manner. Likewise, not only can loss of BRCA1 lead to aneuploidy^44^ but it also contributes to activating the PI3K/Akt signaling pathway[30]. The BRCA1-A complex is important for genomic stability. However, the detailed mechanisms underlying DMs formation for BRCA1 or the BRCA1-A complex deficiency are not completely known. Our results uniquely support the claim that activating the PI3K/Akt pathway induced loss of function of the BRCA1-A complex to cause the production of DMs under the action of Sei-1 in NIH-3T3 cells, and Sei-1 could also target the promoter and regulate the expression of *BRCA1* (Fig. 6). Therefore, the regulation of *BRCA1* was realized in the abovementioned manner. Loss of function of BRCA1 or Abraxas can lead to tumorigenesis^22,33^. Therefore, tumor formation induced by Sei-1 is likely related to the low expression of BRCA1 and Abraxas. On the other hand, genomic instability is also responsible for promoting oncogenesis, which may induce tumor xenografts in Sei-1-transformed NIH-3T3 models.

*Sei-1*, a newly discovered oncogene, is connected to chromosome instability, particularly the formation of DMs, which was the focus of a previous study^4^. The DM population increases with increased passages in vivo but decreases in in vitro cultures due to micronuclei export^5^. Tumors likely adapt to the selective pressure created by the microenvironment through genes carried on DMs. *Met*, *Tspan12*, and *Fam3c*, which are located on DMs, facilitate cancer malignancies, and are correlated with poor prognosis^45–47^. Therefore, the Sei-1-transformed NIH-3T3 model mimics therapeutic approaches that reduce the population of DMs to treat DM-containing tumors. Here, the inhibition of the PI3K/Akt/BRCA1-A complex pathway may serve as an effective measurement to assess therapeutic treatments in Sei-1-associated and DM-containing tumors.

In this study, significantly increased expression of Sei-1 was found using qPCR and immunoblot analysis, which showed that Sei-1 acts as a promoter in accelerating the production of DMs in vivo. In summary, our results revealed a DSB repair defect and that the PI3K/Akt/BRCA1-A complex pathway plays a key role in the formation of DMs. It is necessary to do further studies on the regulatory mechanisms between *Sei-1* and *BRCA1*. Moreover, DM production needs to be explored further by establishing Sei-1-relevant models to extend our understanding of DM evolution under the pressure of microenvironments.

## Materials and methods

### Cell line

NIH-3T3, a mouse embryonic fibroblast cell line, and 293T cells were purchased from the American Type Culture Collection (ATCC, Manassas, VA, USA) and authenticated by means of the STR (short tandem repeat) test (Microread, Beijing, China). The detailed procedures of cell culture, cell passage, and transfection were previously reported^5^.

### Antibodies and reagents

The following antibodies were used for the experiments: anti-BRCA1 and PTEN antibodies (Santa Cruz Biotechnology, Dallas, TX, USA); anti-Rad51, Chk1, and Abraxas antibodies (Abcam, Cambridge, UK); anti-PI3K, pan-Akt, phospho-Akt (Ser473), phospho-Akt (Thr308), and anti-γH2AX antibodies (Cell Signaling Technology, Danvers, MA, USA); IRDye 800DX-conjugated affinity-purified anti-mouse IgG and IRDye 700DX-conjugated affinity-purified anti-rabbit IgG (Rockland Immunochemicals, Gilbertsville, PA, USA); and anti-mouse IgG/HRP and anti-rabbit IgG/HRP secondary antibodies (ZSGB-bio, Beijing, China). The MK2206 2HCl inhibitor (Selleckchem, Houston, TX, USA) was also used.

### Metaphase spread preparation

Cells in metaphase reached 70–80% confluence and were treated with Colchicine (Sigma-Aldrich, St. Louis, MO, USA) for 1.5 h. The cells were trypsinized and then washed with phosphate-buffered saline (PBS) and centrifuged. The cell precipitate was re-suspended with 0.075 M KCl and incubated in a 37 °C water bath for 13 min. After undergoing fixation three times, the cell suspension was dropped on an ice-cold clean slide. The drops spread evenly and were stained with Gimsa (YuanMu, Shanghai, China). The pictures were then photographed using an Olympus BX41 microscope (Melville, NY, USA) equipped with a JVC TKC75U color video camera (JVC, Yokohama, Japan).

### Semi-quantitative and quantitative real-time PCR

Cell precipitates were treated with Trizol (Invitrogen, Carlsbad, CA, USA) to extract the RNA. cDNA samples were obtained using the Transcriptor First Strand cDNA Synthesis Kit (Roche, Mannheim, Germany). Semi-quantitative and quantitative real-time qPCR were performed as previously reported^5,48^.

### Western blot

Cells were lysed with RIPA buffer and centrifuged at 12,000 × *g* at 4 °C for 30 min to extract the total protein. The protein samples were separated on a sodium dodecyl sulfate-polyacrylamide gel electrophoresis gel and transferred to a PVDF membrane. After blocking with 5% blocking buffer, the membranes were incubated with the appropriate antibodies and then scanned using the Odyssey Imaging system (Li-COR, Lincoln, NE, USA) or the Fluorchem R system (FluorChem R, Santa Clara, CA, USA). ImageJ software was used for band analysis.

### Immunofluorescence

The cells covered 80–90% of the glass coverslips. The cells were washed three times with PBS and fixed in 4% paraformaldehyde (Boster, Wuhan, China). Then, the cells were permeabilized with 0.1% Triton X-100 and blocked in 4% bovine serum albumin. Images were obtained using a DM-RXA2 fluorescence microscope (Leica) after incubation with γH2AX antibody (Merck Millipore, Darmstadt, Germany), a fluorescence-conjugated secondary antibody, and DAPI (4′,6-diamidino-2-phenylindole) stain (Helixgen, Guangzhou, China).

### RNA-sequencing

Primary cells were cultured from mouse xenografts as previously described^5^. The cells were sent to the Novogene Company (Beijing, China) for RNA-seq and analysis. We then independently screened, analyzed the data by R (Windows 64 bits, 3.4.2), and validated the DEGs.

### Luciferase reporter assay

The construction of Sei-1 vectors (EX-A3731-Lv103) and pGL3-BRCA1-promoter luciferase reporter vectors were completed by GeneCopoeia company (Guangzhou, China) and Aizhe Biological Technology Co., Ltd (Guangzhou, China), respectively. The 293T cells were plated in 24-well plates at 50,000/well. The co-transfection of Sei-1 and luciferase reporter vectors were accomplished according to the instruction of HilyMax reagents (Dojindo, Shanghai, China). The detection of luciferase activity was based on the instruction of Luc-Pair Duo-Luciferase Assay Kits 2.0 (GeneCopoeia, Rockville, MD, USA) and completed by microplate reader (Molecular Devices, Shanghai, China).

### Statistics

Two-tailed Student’s *t* test and one-way analysis of variance with the Student–Newman–Keuls test were employed for data analysis of DM counts, relative mRNA expression, luciferase reporter assay, and western blots. All data are presented as the mean ± S.D or mean ± S.E.M. Significant differences were defined as *P* < 0.05.

## Electronic supplementary material

Figure legends of supplementary materials

Supplementary figure 1

Supplementary figure 2
