# Supplementary material for: Sei-1 promotes double minute chromosomes formation through activation of the PI3K/Akt/BRCA1-Abraxas pathway and induces double-strand breaks in NIH-3T3 fibroblasts
Source: Cell Death Dis. 2018 Mar 1;9(3):341. doi: 10.1038/s41419-018-0362-y (PMC5832785; doi:10.1038/s41419-018-0362-y)
Supplement: Supplementary file 1 — Figure legends of supplementary materials [file 41419_2018_362_MOESM1_ESM.doc]

**Supplementary Figure Legends**

**Supplementary figure 1.** The analysis of RNA-seq data. (A) The heatmap shows the DEGs between samples C1 (CPX1) and C2 (CPX6). (B) The enrichment analysis of DEGs based on GO and KEGG database.

**Supplementary figure 2.** Sei-1 can target the promoter of *BRCA1*. Luciferase reporter results indicate the luciferase activity is upregulated in Sei-1 and pGL3- BRCA1-promoter co-transfected group compared with the Sei-1 and pGL3-basic co-transfected control group. ***P* < 0.01.
