# Supplementary figures and images for: Sei-1 promotes double minute chromosomes formation through activation of the PI3K/Akt/BRCA1-Abraxas pathway and induces double-strand breaks in NIH-3T3 fibroblasts
Source: Cell Death Dis. 2018 Mar 1;9(3):341. doi: 10.1038/s41419-018-0362-y (PMC5832785; doi:10.1038/s41419-018-0362-y)

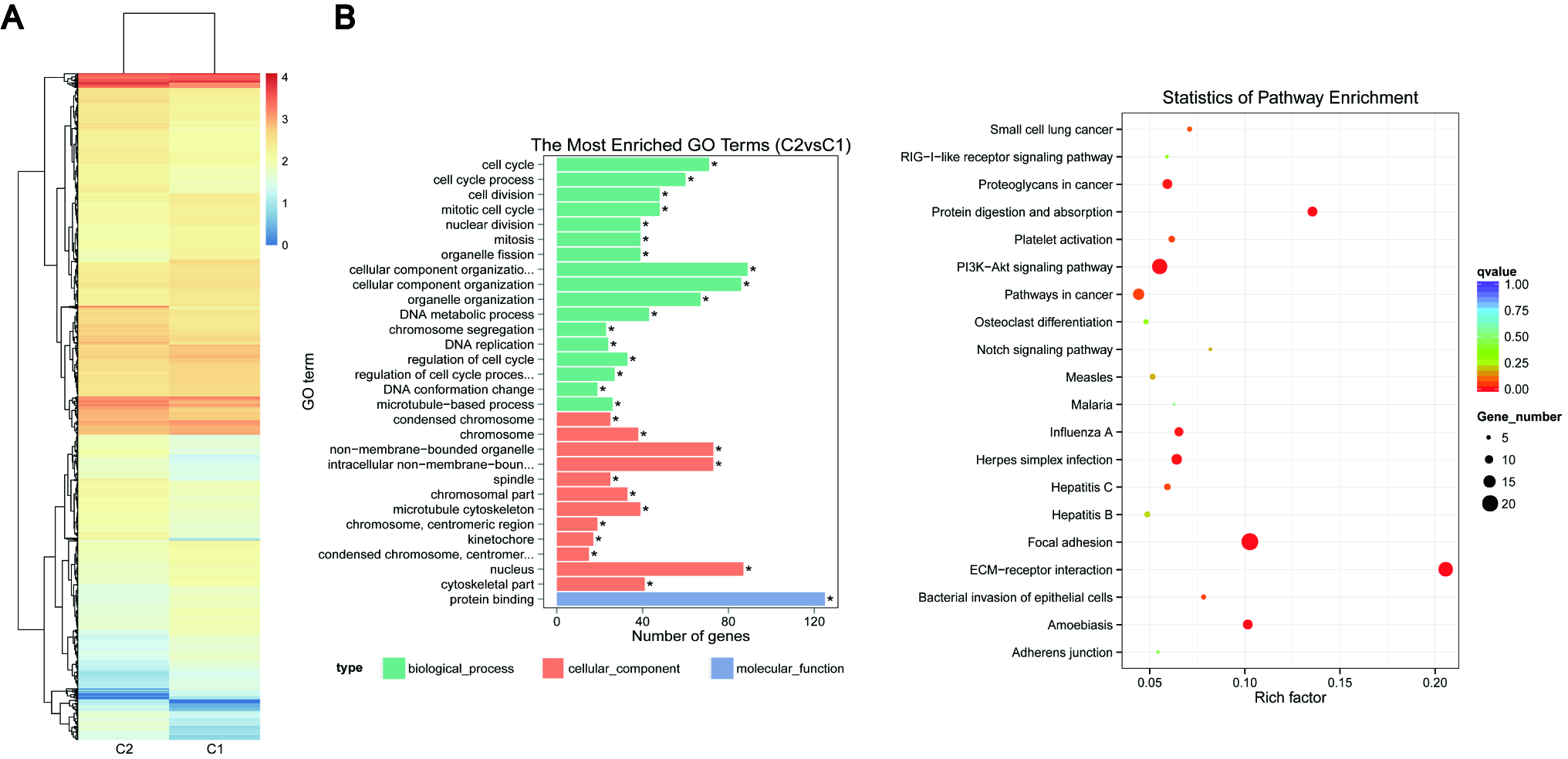

Supplement: Supplementary file 2 — Supplementary figure 1 [file 41419_2018_362_MOESM2_ESM.tif]

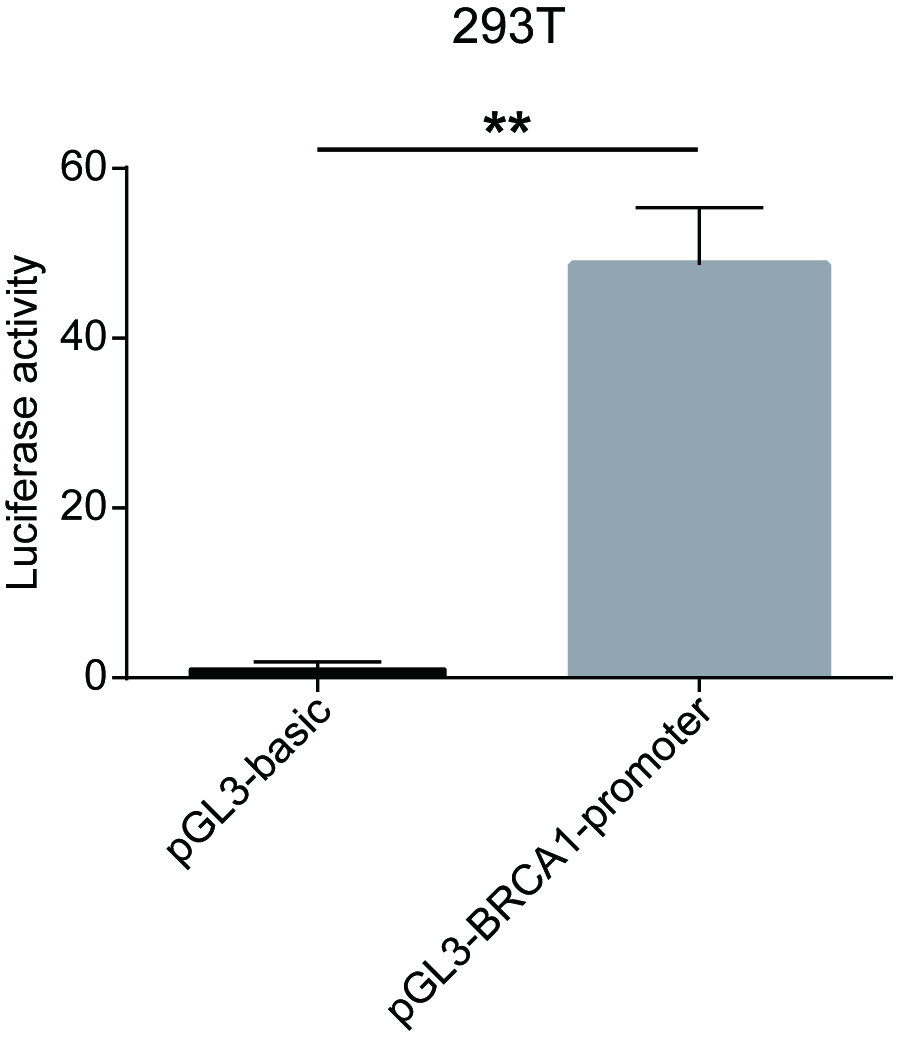

Supplement: Supplementary file 3 — Supplementary figure 2 [file 41419_2018_362_MOESM3_ESM.tif]
